# Role of RNA Polymerase II Promoter-Proximal Pausing in Viral Transcription

**DOI:** 10.3390/v14092029

**Published:** 2022-09-13

**Authors:** Marilyn Whelan, Martin Pelchat

**Affiliations:** Department of Biochemistry, Microbiology and Immunology, Faculty of Medicine, University of Ottawa, Ottawa, ON K1H 8M5, Canada

**Keywords:** RNA polymerase II, promoter-proximal pause, HIV, IAV, HDV, NELF, DSIF

## Abstract

The promoter-proximal pause induced by the binding of the DRB sensitivity-inducing factor (DSIF) and the negative elongation factor (NELF) to RNAP II is a key step in the regulation of metazoan gene expression. It helps maintain a permissive chromatin landscape and ensures a quick transcriptional response from stimulus-responsive pathways such as the innate immune response. It is also involved in the biology of several RNA viruses such as the human immunodeficiency virus (HIV), the influenza A virus (IAV) and the hepatitis *delta* virus (HDV). HIV uses the pause as one of its mechanisms to enter and maintain latency, leading to the creation of viral reservoirs resistant to antiretrovirals. IAV, on the other hand, uses the pause to acquire the capped primers necessary to initiate viral transcription through cap-snatching. Finally, the HDV RNA genome is transcribed directly by RNAP II and requires the small hepatitis *delta* antigen to displace NELF from the polymerase and overcome the transcriptional block caused by RNAP II promoter-proximal pausing. In this review, we will discuss the RNAP II promoter-proximal pause and the roles it plays in the life cycle of RNA viruses such as HIV, IAV and HDV.

## 1. Promoter-Proximal Pausing during RNA Polymerase II Transcription

Transcription of protein-coding genes by RNA polymerase II (RNAP II) is a key step at which gene expression is regulated in eukaryotic cells. Transcription by RNAP II is initiated by the recognition of the core DNA promoter and recruitment of general transcription factors to assemble the preinitiation complex (PIC). Active promoters are suggested to be first recognized by TFIID through its TAF1 and TAF3 subunits, which recognize H4 acetylation and H3K4me3 histone marks, respectively [1,2]. Then, the TATA-binding protein (TBP) subunit of TFIID interacts with the TATA element around 30 bp upstream of the transcription start site (TSS) [3,4,5,6]. Recruitment of TFIIA and TFIIB further stabilizes the TBP-core promoter complex [7,8,9]. RNAP II, complexed with TFIIF, is recruited to the core promoter with the help of TFIIB, which also plays an important role in its correct positioning on the promoter [9,10,11]. The Mediator complex is then recruited to the C-terminal domain (CTD) of the largest subunit of RNAP II (i.e., Rpb1) and serves as a hub for signal transmission to the polymerase from various gene-specific transcriptional activators [12]. The RNAP II CTD, composed of 52 “YSPTSPS” repeats, couples RNAP II transcription and RNA processing events by binding factors required for mRNA processing. The double-stranded DNA at the core promoter is then unwound through the helicase activity of the XPB subunit of TFIIH, which is stabilized and activated by TFIIE. This creates a “bubble” of about 14 nucleotides around the promoter where RNA synthesis can begin [13]. Then, the kinase subunit of TFIIH, CDK7, phosphorylates serine 5 (Ser5-P) of the heptad repeat of the RNAP II CTD. This modification leads to a decreased affinity of the Mediator complex for RNAP II and recruitment and stimulation of transcription factors important for elongation of transcription, such as the capping enzyme [14,15].

**Figure 1:**
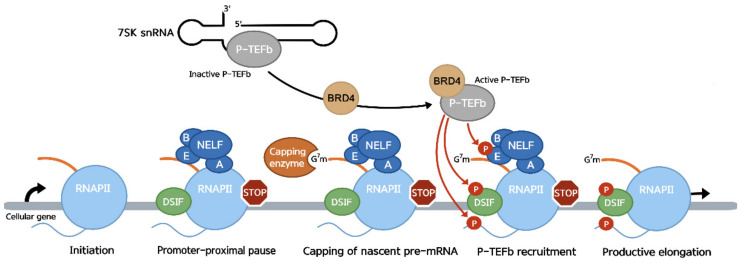
Transcription by RNAP II is paused after initiation as DSIF and NELF are recruited to the polymerase. This pause creates a hub for the capping enzyme, allowing the efficient capping of the nascent transcript. Interaction between P-TEFb and either BRD4 or SEC allows its activation and dissociation from the 7SK snRNP, where it is sequestered in its inactive state. The BRD4- or SEC-P-TEFb complexes are then recruited to the stalled polymerase where the kinase subunit of P-TEFb, CDK9, phosphorylates Spt5, NELF-E and the RNAP II CTD at Ser2. This leads to the dissociation of NELF from the polymerase, the transformation of DSIF into a positive elongation factor and entry into productive elongation.

Soon after initiation, RNAP II pauses mRNA synthesis on a large fraction of transcribed genes, a process called promoter-proximal pausing (Figure 1). Promoter-proximal pausing of transcription by RNAP II is a common regulatory mechanism observed on most metazoan genes. It allows efficient capping of nascent transcripts, and correctly capped nascent mRNA is a prerequisite for escape from the pause [16,17,18,19]. It also allows maintenance of a permissive chromatin landscape around active promoters and a quick transcriptional response from stimulus-responsive genes involved in pathways such as the innate immune response and cell cycle [20,21]. The pause occurs shortly after promoter clearance of RNAP II following the recruitment of negative elongation factors to the transcribing polymerase [22,23]. The DRB sensitivity-inducing factor (DSIF), a heterodimer composed of Spt4 and Spt5, is the first to interact with RNAP II after promoter clearance. The interaction between Spt5 and RNAP II slows the processivity of the polymerase [24,25], but transcription is fully paused only once the negative elongation factor (NELF) is recruited to the transcriptional complex. NELF, a protein complex composed of NELF-A, -B, -C/D and -E, interacts with both RNAP II and the nascent transcript. NELF is thought to be first recruited to the pause site through recognition of the NELF-E binding element (NBE) by the RNA recognition motif (RRM) in its NELF-E subunit. NELF-A then binds the DSIF-RNAP II complex and fully halts transcription elongation [22,26,27].

The paused RNAP II remains stably associated with both the nascent pre-mRNA and DNA template and resumes elongation when stimulated by the kinase activity of the positive elongation factor P-TEFb, a heterodimer composed of cyclin-dependent kinase 9 (CDK9) and cyclin T [28,29,30]. The majority of cellular P-TEFb is sequestered and inactivated by the 7SK snRNP complex [29,30,31]. Interactions with either the super elongation complex (SEC) or BRD4 activates P-TEFb, leading to its dissociation from the 7SK snRNP complex and its recruitment to the paused RNAP II [32]. SEC- or BRD4-P-TEFb complexes then phosphorylate the RNAP II CTD, Spt5 and NELF-E [32,33,34]. The RNAP II CTD is phosphorylated at the serine 2 (Ser2-P) residues of the heptapeptide, creating a platform for binding RNA-processing factors and facilitating productive RNA synthesis. Phosphorylation of NELF-E induces its dissociation from the polymerase, while phosphorylation of Spt5 effectively transforms DSIF into a positive elongation factor, stimulating RNAP II processivity [28,35].

RNAP II pausing is a widespread strategy for governing transcription output and mRNA processing. DSIF is known to stimulate recruitment and guanylylation activity of the capping enzyme on the nascent transcript [16,17,18], while NELF is known to interact with the cap-binding complex to further stabilize the capped transcript [19]. The pause not only serves as a transcription brake to modulate gene expression but also prevents deposition of nucleosomes in promoter-proximal regions of actively transcribed genes, keeping them in an open configuration [21]. Particularly, a significant enrichment of RNAP II/NELF complexes is observed just downstream of promoters of genes involved in signal-responsive pathways, including development, cell proliferation, immunological signaling and stress or damage responses. The presence of paused RNAP II on these genes represents a framework for rapid transcription activation in response to specific cues, and many mammalian genes with paused RNAP II have fast, transient expression kinetics (e.g., JUNB and TNF-alpha) [20,21,36].

As the promoter-proximal pause plays such a key role in transcription regulation, it is not surprising that it is also involved in the pathogenesis of different viruses using RNAP II for their life cycle, such as the human immunodeficiency virus (HIV), the influenza A virus (IAV) and the hepatitis *delta* virus (HDV).

## 2. The Human Immunodeficiency Virus (HIV)

HIV is a retrovirus known to infect CD4+ T cells and macrophages [37,38,39,40], causing a slow depletion of the immune cell pool and eventually leading to the acquired immunodeficiency syndrome (AIDS) if left untreated [41,42,43,44]. Like other retroviruses, HIV integrates a DNA version of its single-stranded RNA genome into a host cell chromosome. The integration process is mediated by the integrase (IN), a viral protein that binds the reverse transcript, processes its 3′ ends, digests chromosomal DNA and joins the viral DNA to the target cellular DNA [45]. This reaction is then completed by host enzymes, which repair the single-stranded break at the integration sites [45]. HIV integration is non-random. The HIV IN was found to favor gene-dense, transcriptionally active regions of chromatin with lower nucleosome density [46,47]. Once integrated, transcription of proviral genes is mediated by the host RNAP II.

**Figure 2:**
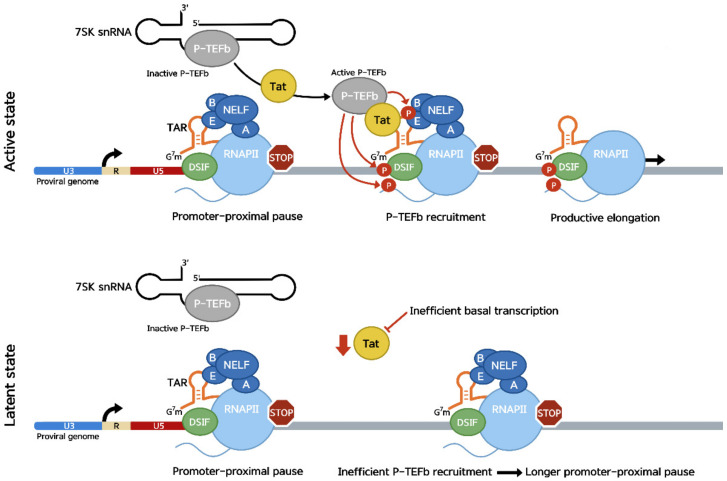
(Active state) Transcription on the provirus is paused shortly after RNAP II transcribes through the TAR element. The interaction between P-TEFb and Tat leads to the activation and dissociation of P-TEFb from the 7SK snRNP, where it is sequestered in its inactive form. Tat-P-TEFb complexes are then recruited to the stalled polymerase through the interaction between Tat and TAR. The kinase subunit of P-TEFb, CDK9, and then phosphorylates Spt5, NELF-E and the RNAP II CTD at Ser2, leading to entry into productive elongation. (Latent state) Low levels of Tat in the infected cell due to insufficient amounts of cellular TFs, promoter mutations and low concentrations of nucleotides impedes basal HIV transcription. Therefore, P-TEFb remains sequestered by 7SK snRNP, and the polymerase remains stalled in a promoter-proximal manner, leading to latency.

In the context of an active infection, HIV transcription is initiated at the 5′ long terminal repeat (LTR), which is divided in three sections: the untranslated 3′ (U3), the repeat (R) and the untranslated 5′ (U5). The LTR U3 region contains binding sites for transcription factors (TFs), such as NF-κB and Sp1 [48]. Through various mutational and in vitro transcription studies using LTR constructs, the binding of these TFs to the LTR was shown to greatly increase transcription initiation on the provirus and are therefore important for the activation of viral transcription [48,49,50,51]. The U3 region also contains a TATA box and initiator element, both important for PIC assembly and initiation of transcription, which occurs at the junction between U3 and R. Transcription on the provirus by RNAP II is then paused by the joined action of NELF and DSIF. The promoter-proximal pause on the provirus occurs shortly after the transactivation response (TAR) element has been transcribed (Figure 2) [35,52,53,54]. The TAR element forms a stem-loop structure in the nascent transcript [55,56,57] and contains an NBE-like motif in its loop. Binding assays showed that this NBE-like motif is required for NELF-E recruitment and its binding to the TAR loop [27]. Viral transcription elongation is then stimulated through recruitment of the trans-activator of transcription (Tat) viral protein to the provirus [55,56,57,58]. Protein affinity chromatography using nuclear extracts found that Tat interacts with the cyclin T subunit of P-TEFb [59]. The high affinity of Tat for P-TEFb allows it to compete with the 7SK snRNP complex [59,60], in which the majority of cellular P-TEFb is sequestered and inactivated [29,30,31]. The binding of Tat to cyclin T leads to the dissociation of P-TEFb from the 7SK snRNP complex and the activation of its kinase subunit, CDK9. The Tat-P-TEFb complex is then recruited to the provirus through the interaction of Tat with the TAR element (Figure 2; active state). Indeed, it was shown that deletion of the bulge region of TAR greatly reduces the Tat-mediated transcriptional stimulation [50]. P-TEFb can then stimulate transcription elongation by phosphorylating Ser2 on RNAP II CTD, NELF-E and Spt5 [35,53,59,61], as described above.

As Tat is not packaged in the virion, early HIV transcription relies on the release of stalled RNAP II into productive elongation using cellular TFs such as NF-κB [51,62] and Sp1, as well as the recruitment of SEC- and BRD4-P-TEFb complexes [63]. In activated T cells, this process efficiently leads to the production of early HIV transcripts, which produce a subset of viral proteins, including Tat [64,65]. In the context of a latent infection, basal HIV transcription is hindered by insufficient amounts of cellular TFs, promoter mutations and low concentrations of nucleotides in the host cell [66,67,68]. As Tat expression is hindered by the low transcriptional rates, P-TEFb is not efficiently recruited to the viral genome, leading to a prolonged promoter-proximal pause (Figure 2; latent state) [44,48,54]. This is one of the mechanisms through which HIV enters latency, a state in which the virus is transcriptionally silent. Other mechanisms leading to latency include proviral integration into heterochromatin and transcriptional interference [69,70]. Latently infected cells create viral reservoirs, which are not targeted by T-cell responses or antiretroviral drugs due to minimal viral RNA transcription and translation [42,71].

Latency not only allows HIV to lie undetected during antiretroviral therapy, but it is also beneficial for productive infection of new hosts and maintenance of said infection. Rouzine et al. used mathematical models tested on patient data to study the evolutionary role of HIV latency [72]. By taking several factors into consideration, including the probability of latency, the probability of establishing a systemic infection and the immune response, they were able to show that although latency reduces the infection inoculum, it greatly increases the probability of establishing a systemic infection as viruses unable to enter a latent state are more likely to be eradicated at the infection site [72]. Therefore, latency is an unavoidable aspect of HIV infections. The problem with latency is its reversibility, as once viral replication is reactivated, the disease progresses. A portion of the viral reservoirs is composed of resting T cells, which will be activated by the recognition of their antigen. This leads to a replenishment of cellular TFs and therefore the reactivation of HIV transcription and replication [73,74,75]. If reactivation of latently infected cells occurs in the presence of antiretroviral drugs, the newly activated infected cells will be targeted and eliminated. If it occurs once treatment has been interrupted, the newly activated infected cells remain and unleash their viral load in the bloodstream, causing a relapse [41,42,43,44].

## 3. The Influenza A Virus (IAV)

Known to infect millions of individuals every year, IAV is an RNA virus that causes a mild-to-severe respiratory disease. IAV is a negative-sense RNA virus with a segmented genome formed by eight single-stranded viral RNA segments (vRNAs). The IAV RNA-dependent RNA polymerase (RdRp) is responsible for replicating each genomic segment, which are then packaged in newly formed virions, and for transcribing the viral genes, which are 5′-capped and 3′-polyadenylated [76,77,78,79,80,81,82,83,84,85,86]. In the nucleus of infected cells, each of the vRNA segments is associated with its own copy of the RdRp, bound at the double-stranded viral promoter formed by a structure composed of the first 12 and 13 nucleotides at the 3′ and 5′ ends of the vRNAs [86,87,88,89]. The RdRp is composed of three subunits: the polymerase basic protein 1 (PB1), the polymerase basic protein 2 (PB2) and the polymerase acidic protein (PA). PB1 possesses the polymerization activity [90,91,92,93], while the other two are involved in initiation of transcription by binding to and cleaving capped host pre-mRNA, respectively [89,90,94,95,96,97,98,99,100,101,102,103]. In addition, IAV nucleoprotein (NP) is part of the RdRp complex by interacting with PB1 and PB2 and binding to the vRNA template [104,105,106]. Once transcribed, viral mRNAs are translated by the host cell machinery.

Although IAV mRNAs are capped like cellular mRNAs, the viral polymerase does not have capping abilities. Instead, the viral RdRp interacts with transcribing RNAP II to bind the 5′ cap structure of nascent pre-mRNAs and, following cleavage, uses capped RNA primers derived from host pre-mRNAs to initiate viral transcription [107,108,109]. This mechanism, known as cap-snatching, is initiated by the recruitment of the IAV RdRp to RNAP II through recognition of Ser5-P on the polymerase CTD, a modification associated with the initiating and paused forms of RNAP II. This interaction is mediated by the PA subunit and has been proposed to promote the opening of the cap-binding domain found in the PB2 subunit [108,110]. Once PB2 has bound the cap structure on the nascent pre-mRNA and the RdRp is properly docked on the RNAP II, the endonuclease domain of the PA subunit cleaves the nascent transcript 10 to 15 nucleotides downstream of the cap structure [96,99,111,112,113,114]. The capped pre-mRNA fragment is then inserted into the catalytic center of PB1 where it is extended using the vRNA as a template [85,107,113,114,115,116,117,118,119,120]. The vRNA templates are required for cap-snatching and host primers with multiple bases complementary to the vRNA templates are preferentially used to initiate IAV transcription [107,111,121,122,123,124]. Approximately 14.3% of the cap-snatched primers are elongated to form four base pairs with the vRNA templates and then realigned at the last 3′ nucleotide of the vRNAs and re-elongated [121]. This process, called prime-and-realign, is used to allow efficient IAV transcription by rescuing most of the suboptimal cap-snatched primers [114,121].

**Figure 3:**
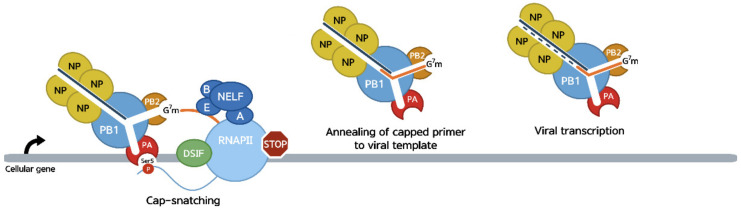
The IAV RdRp interacts with the paused RNAP II through its PA and PB2 subunit. The PA subunit binds Ser5-P on the CTD, opening the cap-binding domain of PB2. Once the cap structure is bound by PB2, the endonuclease domain of PA cleaves the nascent pre-mRNA, which is then inserted in the catalytic center of PB1 and extended using the vRNA as a template.

The IAV RdRp acquires most of its capped primers by interacting with the paused RNAP II (Figure 3). Indeed, peptide pulldown assays using copies of the RNAP II CTD phosphorylated at serine 5 (Ser5-P), typical of the initiating and paused RNAP II, serine 2 (Ser2-P), typical of the elongating polymerase, or left unphosphorylated showed that the IAV RdRp only had a high affinity for the Ser5-P CTD, not for the Ser2-P CTD or its unphosphorylated form [110]. This was further confirmed by ChIP-PCR assays in IAV-infected cells, which showed the RdRp associates preferably with the promoter region rather than with the gene body, leading to depletion of RNAP II on the gene bodies. Furthermore, it was found that while the RdRp activity is sensitive to α-amanitin, a potent RNAP II inhibitor, inhibition of RNAP II elongation using DRB to inhibit P-TEFb activity has no effect on viral transcription by the RdRp [125], indicating that the IAV RdRp does not require the RNAP II to be actively elongating for cap-snatching to occur. These findings suggest that IAV RdRp binds to and inhibits RNAP II elongation at an early stage in the transcription, during promoter-proximal pausing, putting the viral RdRp in an ideal place to specifically access cellular capped primers at the time when RNAP II is pausing and nascent transcripts are being capped, or have just been capped.

Not only does cap-snatching provide the 5′ cap modification necessary for protection of viral mRNAs against 5′-3′ exonucleases and their translation by the cellular machinery, but it also plays a part in the shutoff of cellular gene expression. Indeed, IAV infection is known to deplete RNAP II from the gene bodies, but not from their promoters, efficiently hindering elongation by RNAP II and therefore gene expression [125,126]. It is thought that cap-snatching could be the cause of the reduced occupancy of RNAP II on the gene bodies, as the uncapped pre-mRNAs could be targeted by the cellular exonuclease Xrn2, their degradation leading to the release of RNAP II [109,127]. Furthermore, the RdRp tends to target transcripts based not only on their complementarity to the vRNA [111,114,121,122] but also on their level of transcription, and highly abundant RNA, such as small nuclear RNAs, could be favored as cap-snatched host primers [114,128]. It has been proposed that RNAP II depletion and cap-snatching of the most abundant host mRNAs could globally modulate the expression of host genes and contribute to host shut-off early after infection [114,121]. Furthermore, it is likely that genes involved in the antiviral responses would be more likely to be targeted for cap-snatching, as they are upregulated upon infection. This would hinder the cellular antiviral response and give the virus the advantage it needs to overtake the cellular machinery. Because of the implication of RNAP II promoter-proximal pausing in IAV transcription and probably in host shut-off, it is possible that reduction of RNAP II pausing, by NELF depletion or inhibition, could reduce cap-dependent viral transcription and induce the antiviral responses, which might be an approach for an antiviral therapy against this highly contagious virus.

## 4. The Hepatitis *delta* Virus (HDV)

HDV is a satellite virus of the hepatitis B virus (HBV). While HDV depends on HBV to propagate, both viruses replicate independently from one another in infected cells, and they share no sequence similarity [129]. HDV has a ~1,680 nucleotides long circular, single-stranded RNA genome able to adopt an unbranched rod-like structure due to 74% self-complementarity [130]. This structure is proposed to stabilize the HDV genome from degradation by host exonucleases and endonuclease by preventing their easy access to cleavage sites [131]. The HDV genome contains a single open reading frame (ORF), coding for two viral proteins: the small and the large antigen (HDAg-S and HDAg-L). HDAg-S is produced earlier in the infection and is essential for HDV accumulation. Later in the infection, the host adenosine deaminase that acts on RNA (ADAR-1) edits some antigenomic HDV RNA genomes at a location corresponding to the termination codon of the HDAg-S gene, and the resulting HDV genomes produce HDAg-L, which contains an additional 19 amino acids at its C-terminus [132]. HDAg-L is required for virion packaging and was also reported to negatively affect HDV accumulation when provided before replication [133,134]. In addition, both genomic and antigenomic polarities of the HDV genome possess a self-cleaving ribozyme domain essential for virus replication.

HDV replicates via a symmetrical, rolling circle mechanism. Replication from the circular monomer of genomic RNA produces linear, multimeric antigenomic strands. These multimers self-cleave to monomers by endogenous ribozymes and are then ligated, to produce antigenomic circular monomers. These antigenomic molecules then serve as templates for genomic RNA synthesis using the same steps. In addition, HDV genomes of genomic polarity serve as templates for the synthesis of the HDAg mRNA. HDV does not encode its own RdRp and does not reverse transcribe into DNA intermediates [135]. HDV replication and transcription take place in the nucleus of infected cells. HDAg mRNA is post-transcriptionally processed with a 5′-cap and a 3′-poly(A) tail, which are typical features of transcripts generated by RNAP II [136,137]. HDV RNA accumulation in cultured cells is also sensitive to low doses of α-amanitin, at concentrations typical for RNAP II inhibition [138]. RNAP II and the TATA binding protein (TBP) associate with HDV RNA, both in cells replicating HDV RNA and in vitro [139,140]. Furthermore, RNA affinity chromatography, using an HDV RNA promoter and nuclear extract, established that an active RNAP II preinitiation complex forms on the RNA promoter, similar to what is typically observed on DNA promoters, containing the core RNAP II subunit and the general transcription factors TFIIA, TFIIB, TFIID, TFIIE, TFIIF, TFIIH and TFIIS [139]. Binding assays using purified proteins demonstrated the direct binding of the TATA-binding protein and suggested that, as part of TFIID, it is required to nucleate the RNAP II complex on the HDV RNA promoter [139]. Because antigenomic RNA synthesis is more resistant to α-amanitin, the involvement of at least one other host RNAP in HDV replication has been proposed [141,142,143]. Consistent with this hypothesis, RNAP I and RNAP III can associate with both polarities of HDV RNA, both in cells replicating HDV RNA and in vitro, and RNAP I co-localizes with HDV antigenomic RNA [141,144]. Moreover, the RNAP-I-specific transcription factor SL1 can be co-immunoprecipitated with HDAg, and inhibition of SL1 activity resulted in an 80% reduction of antigenomic RNA synthesis [141].

**Figure 4:**
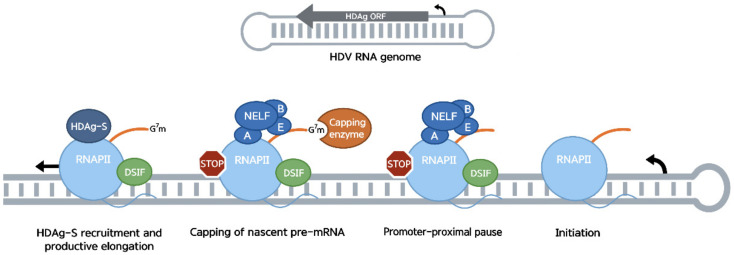
RNAP II initiates transcription on the HDV RNA genome. Transcription is paused ~40 nucleotides after initiation, following recruitment of DSIF and NELF to the polymerase. Once the nascent transcript is capped, HDAg-S is recruited to the stalled complex where it competes with NELF-A for a common binding site on RNAP II. HDAg-S displaces NELF from RNAP II, stimulating processivity of the polymerase and therefore entry into productive elongation.

Although the role of RNAP II in both HDV replication and transcription is well established [135,139,140,145,146,147], the mechanism for such a template shift for RNAP II remains mostly unclear. However, because both the sequences and the highly conserved rod-like conformation of the terminal stem-loops were shown to play important roles as HDV promoters, such features were proposed to facilitate recruitment of the transcriptional complex on the RNA genome [139,140,148]. While the transcriptional complex can be recruited to the HDV genome without further stimulation, in vitro transcription using HDV RNA templates was observed to stop ~40 nucleotides after initiation [146,149]. This location is consistent with an event involving the recruitment of DSIF and NELF to RNAP II and promoter-proximal pausing. Interestingly, HDAg-S binds directly to the clamp module of RNAP II, formed by Rbp1 and Rbp2 [150]. This interaction accelerates forward translocation of RNAP II and affects its transcriptional fidelity [26,150,151]. This is thought to be due to a loosening of the polymerase clamp, which may affect its ability to recognize incoming bases as well as the template. The conserved C-terminus region of HDAg-S has a weak sequence similarity (~27% identity) with the N-terminal region of NELF-A involved in RNAP II binding, extending to their predicted secondary structures [26,149]. Using in vitro transcription assay with nuclear extract and both DNA and HDV-derived RNA templates, HDAg-S was shown to reverse DRB inhibition and to counteract the negative effect of DSIF/NELF [149]. In addition, HDAg-S binding to RNAP II inhibits NELF-RNAP II association and stimulates RNAP II elongation in a DSIF/NELF-independent manner. Deletion of the HDAg-S C-terminus region impairs this effect, consistent with competition for a common binding site between HDAg-S and NELF-A on RNAP II [149]. It is therefore proposed that HDAg-S stimulates transcription elongation of the viral genome by reducing promoter-proximal pausing through displacement of NELF from the transcriptional complex (Figure 4). As such, it is possible that HDAg-S affects not only HDV RNA transcription and replication but also host transcription and might have a role in this virus’s pathogenesis. Consequently, increasing RNAP II pausing on HDV RNA templates, by upregulating NELF or through synthetic molecules inhibiting the binding of HDAg-S to RNAPII, might be an approach to disrupt both HDV transcription and replication. Furthermore, using HDAg-S as a NELF inhibitor in studies looking into the RNAP II promoter-proximal pausing and its effect on gene expression could compensate for the lack of NELF inhibitors currently available. Its use as a NELF inhibitor could also be useful as part of antiviral therapies against viruses that use the promoter-proximal pause during their life cycle, such as the ones described in this review.

## 5. Conclusions

The promoter-proximal pause is a key step in the regulation of metazoan gene expression. Not only does it prevent deposition of nucleosomes on active promoters, but it also allows a quick response to stimuli from genes involved in signal-responsive pathways such as the innate immune response and cell proliferation. Transcription pausing and entry into productive elongation relies on the interplay between RNAP II and negative and positive elongation factors such as DSIF, NELF and P-TEFb. Recruitment of the negative elongation factors also creates a hub for the capping enzyme to add the 5′ cap to the nascent pre-mRNA. The promoter-proximal pause is also important for the pathogenesis of many different viruses such as HIV, IAV and HDV. The pause is one of the mechanisms through which HIV enters latency and maintains its dormancy in immune cells. These latently infected cells form viral reservoirs that remain dormant until treatment is interrupted, at which point viral replication restarts and the disease progresses. These latently infected cells are standing in the way of a sterilizing cure for HIV. In the case of IAV, the viral RdRp requires capped primers to initiate viral transcription, and it acquires those by interacting with the paused RNAP II and its nascent capped pre-mRNA. Following cap-snatching, the cellular pre-mRNA is degraded by exonuclease Xrn2 and RNAP II is then released, leading to a depletion of RNAP II from gene bodies and a reduction in cellular gene expression. As such, cap-snatching is proposed to play a role in the host response shut off, as the RdRp tends to target transcripts not only based on their complementarity to the template vRNA but also on their abundance. As genes involved in the innate immune response are upregulated upon infection, they are more likely to be targeted by the IAV RdRp. As for HDV, its circular single-stranded RNA is able to recruit RNAP II and the other TFs necessary to initiate transcription. Although the mechanism for such a template switch remains elusive, studies have shown that the polymerase can initiate from the RNA promoter situated at the terminal stem-loops of the viral genome. Although RNAP II can initiate transcription on the HDV genome, it requires HDAg-S to enter productive elongation. Due to a weak sequence similarity to NELF-A, HDAg-S is proposed to reduce the promoter-proximal pause on the HDV genome by displacing NELF from the transcriptional complex and stimulating RNAPII processivity.

This review has focused on RNA viruses, but some DNA viruses are also known to use or affect the promoter-proximal pause during infection, such as the herpes simplex virus 1 (HSV-1). HSV-1 is known to shut off host gene expression through various mechanisms, one of which is proposed to be associated with a loss of Ser2-P RNAP II during the infection [152,153]. HSV-1 also takes advantage of the promoter-proximal pause to regulate viral gene expression. It was shown that the promoter-proximal pause increased on early genes between 3 and 6 hours post infection, effectively reducing their expression and allowing the intermediate genes to take over [154].

Disrupting the promoter-proximal pause is a very ingenious way of regulating gene expression. As genes involved in the innate immune response are regulated at the promoter-proximal region, disruption of the pause allows viruses to repress the cellular immune response during viral replication. Using the pause is also a great way to regulate gene expression in a temporal fashion so that genes are expressed in a specific sequence. As such, regulating RNAP II transcription, specifically the pausing, could be a very interesting avenue for future antiviral therapies against pathogens known to use or disrupt the RNAP II promoter-proximal pause as part of their replication cycle.

## Data Availability

Not applicable.
